# A sample holder system for high-precision and reliable resistivity measurements of laboratory lime-cement samples

**DOI:** 10.1016/j.ohx.2026.e00795

**Published:** 2026-05-20

**Authors:** Mikael Lumetzberger, Torleif Dahlin, Tina Martin, Per Hedblom, Simon Rejkjær, Per-Ivar Olsson

**Affiliations:** a Engineering Geology, Lund University, Sweden; b WSP, Sweden

**Keywords:** Lime-cement, Resistivity, Method development, Laboratory

## Abstract

Lime-cement mixing is a method used for ground improvement of clay soils. Before mixing at a field site, laboratory samples are prepared and tested to decide the amount of lime-cement binder needed to reach the desired material strength. Strength measurements are not possible before a sample hardens, and the test process to predict final strength takes 30 days. Resistivity measurements are possible immediately after sample preparation. If a correlation can be made between lime-cement resistivity and strength, resistivity measurements could provide a useful early indication of sample strength development. Laboratory resistivity measurements have shown a correlation between resistivity and material strength in cement-based mixtures, but lime-cement mixtures have not been thoroughly investigated.

Conventional four-electrode soil box sample holders are not well suited to resistivity measurements of laboratory-prepared lime-cement samples, since the rectangular shape makes packing and removal of the material impractical. The method is also inherently destructive due to spike electrodes that penetrate the sample, which would bias strength measurements. Non-intrusive two-electrode methods can be used but they introduce measurement uncertainties. Additionally, the hydration of lime-cement is an exothermic reaction. To quantify the effect of temperature variations on measured resistivity, sample temperature measurements are needed.

We present a new sample holder design with non-intrusive sensors that enables temperature compensated 4-electrode resistivity measurements of laboratory-prepared lime-cement samples. Sensors are integrated into 3D-printed lids that comply with standard geotechnical sample cylinder dimensions for ease-of-use with a high repeatability (<0.2% coefficient of variation).

## Highlights

• Novel lid design enables non-intrusive resistivity lab measurements.
• Sensors (4 electrodes and 1 temperature) are integrated into each lid.
• Compatible with standard geotechnical sample cylinders used in labs.
• 8-sample setup for automated measurements.
• Effective integration into existing geotechnical lab workflows.

## Graphical abstract

**Figure:**
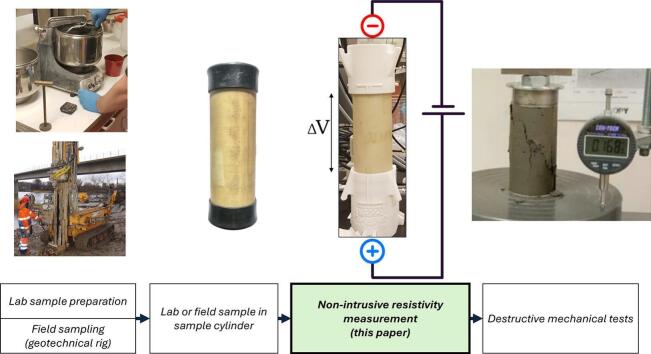

**Specifications table:** 

Hardware name	Cylindrical sample holder system with integrated electrodes
Subject area	Engineering and materials science
Hardware type	Measuring physical properties and in-lab sensors
Closest commercial analog	Resistivity soil box [1]
Open source license	*CC BY 4.0*
Cost of hardware	57900$ for 8 sample holders (7900$ excluding ABEM Terrameter LS2)
Source file repository	https://doi.org/10.17605/OSF.IO/9AYVT

## 1 Hardware in context

Previous studies have investigated the hardening of cement and the relationship between material strength and resistivity in cement-based mixtures [2], [3], [4], but not for lime-cement. The relationship is of interest as resistivity could provide early information about strength development (before the lime-cement paste hardens). Lime-cement mixing is a ground improvement method where binder is injected into and mixed with in-situ clay. Prior to mixing at a field site, laboratory samples are prepared and tested to determine the amount of binder needed to achieve the desired material strength (a process that requires 28 days to provide a reliable prediction of long-term strength). After mixing the ingredients (clay, lime and cement) into a homogeneous paste, sample material is tamped into cylindrical sample holders and left to cure. The exothermic curing reaction involves hydration, and samples are sealed to prevent atmospheric moisture exchange from affecting the reaction. Unconfined compressive strength (UCS) tests can begin when hardening starts, after c:a 7 days of curing. Consecutive UCS tests are done at intervals to follow the strength growth and to predict long-term strength. Since UCS tests are destructive, batches of samples are prepared together.

Sample holder designs for lab resistivity measurements are conventionally based on the two-electrode or the four-electrode soil box methods [5], [6], although several non-proprietary designs exist for various applications [7], [8], [9]. Conventionally, a sample is packed into a rectangular box with two metal plate electrodes at the ends for current injection and two potential electrodes (metal spikes) that penetrate the box sides and the sample. The soil box is unsuitable for the purpose of investigating the relationship between lime-cement resistivity and strength, since the penetrating electrodes affect the results of mechanical tests and the box geometry makes removal of hardened samples difficult without breaking the brittle samples. Non-penetrating two-electrode designs are sometimes used [6], [10], [11] but this method introduces uncertainty since it uses the same electrodes for current transmission and potential measurements. Therefore, the contact resistance at the interface between the electrodes and sample material can have a large impact on the measured potential, without any possibility to quantify it, which is avoided using four-electrode measurements. These limitations, and large variations in methodology and proprietary sample holder dimensions, limit the reproducibility and repeatability of laboratory resistivity measurements.

Here, we present a new sample holder design that enables high quality non-intrusive resistivity measurements of laboratory prepared lime-cement samples, together with a practical methodology with a high repeatability. Sensors are integrated into 3D-printed sample holder lids that comply with standard geotechnical sample cylinder dimensions for efficient measurements. A practical application of the design is shown in [12], where the sample holder system was used to investigate the relationship between and resistivity and unconfined compressive strength in curing dredge mass stabilization samples. Large amounts of high-quality data were collected for the study and same-sample comparison between compressive strength and resistivity could be made without bias due to our non-intrusive sensor design.

## 2 Hardware description

### 2.1 Design in short

Our new non-intrusive sensor design streamlines the measurement workflow, allowing efficient collection of lab prepared lime-cement sample resistivity data with high repeatability. Temperature compensation and a standard sample form factor reduce bias when making cross-study and cross-laboratory comparisons.

### 2.2 Highlights

• Novel lid design compatible with standard geotechnical sample cylinders enables non-intrusive resistivity measurements of lab prepared samples.
• Resistivity measurements can easily be made on the same sample used for other test methods (e.g. mechanical tests).
• Sensors (4 electrodes and 1 temperature) are integrated into each lid.
• Lids are attached to standard cylinder sample containers used in soil sampling tools and geotechnical labs [13], sealing the lime-cement sample to prevent drying into the air.
• Temperature compensation of resistivity data is made possible, removing temperature effects on measured resistivity [14].
• Multi-sample setup allows effective integration into existing lab workflows and automatic measurements (depending on resistivity meter, in the present paper up to eight samples).

### 2.3 Measurement system components

• Sample cylinder containing sample material
• Pair of lids with integrated electrodes and temperature sensors. Lids attach to sample cylinder
• Resistivity meter
• Temperature logger
• Mini-PC

**Fig. 1:**
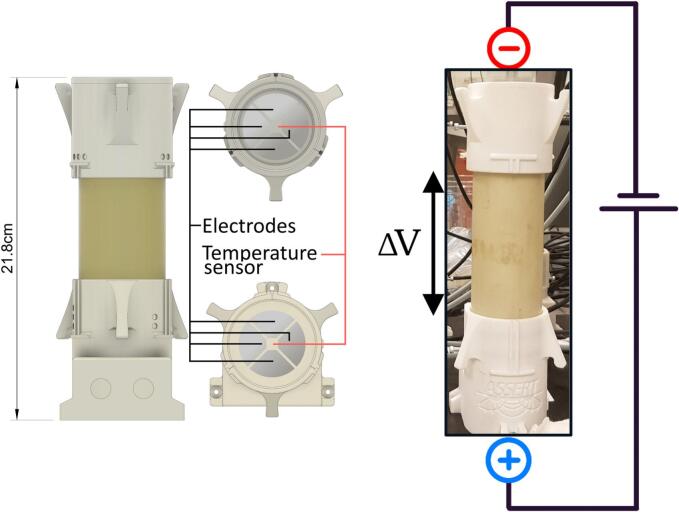
3D drawings of the lids (without cables) and a photo that shows the top and bottom cylinder lids attached to a sample cylinder, ready for electrical measurement. The pie shaped electrodes in the lids are used to inject current through the sample and to measure the resulting potential. The temperature sensor is embedded in the central square space between the electrodes.

**Fig. 2:**
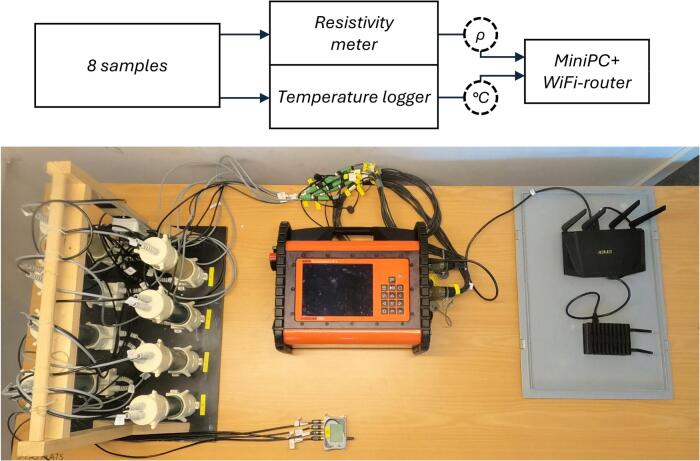
The 8 sample holders to the left in the photo connect directly to the Temperature logger and through two cable assemblies to the 32 pin ports on the Resistivity meter. The Resistivity meter and Temperature loggers are then controlled by the Mini-PC through the network of the Wi-Fi Router. The flowchart (top) shows the data connections between the system components displayed in the photo. The flowchart circles show the data flow of resistivity (ρ) and temperature (°C).

A pair of lids and an empty sample cylinder are shown in Fig. 1. A full set-up of all components, with eight samples, is shown in Fig. 2.

### 2.4 Resistivity measurement theory

A material’s electrical resistivity *ρ* is calculated from measurements of the potential difference Δ*U* between two electrodes during current injection *I, using* the equation *ρ = k∙*Δ*U/I.*

**Fig. 3:**
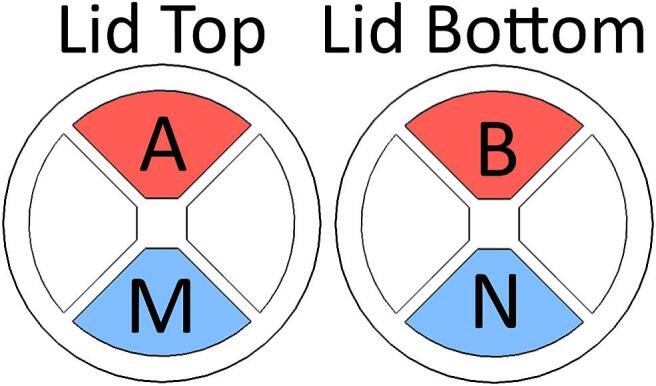
Illustration of the relative positioning between current (A,B) and potential (M,N) electrodes in the lids during measurement. Current is transmitted through the sample material between the top and bottom lid with AB electrodes positioned on top of each other. The MN electrodes used for potential measurements are on top of each other and opposite to the AB current electrodes. This electrode array can be rotated in four steps to give an indication of sample heterogeneity.

The geometric factor *k* depends on the relative positioning and shape of the current and potential electrodes. Fig. 3 describes the electrode array used for measurements. Assuming that potential electrodes are spaced apart length L in the direction of a homogenous current flow, if two samples differ in length the relation between *k_1_* of sample 1 and *k_2_* of sample 2 is *k_1_∙L_1_ = k_2_∙L_2_*. If a value for *k_1_∙L_1_* is known (e.g. by calibrating the sample holder using a solution of known resistivity) and *L_2_* is measured, then *k_2_ = k_1_∙L_1_/L_2_.*

The resistivity is temperature dependent: *ρ* measured at the temperature *T* can be compensated with the temperature correction factor *f_t_* to the equivalent resistivity at 25 °C (i.e. *ρ_25°C_)* with *ρ_25°C_* = *ρ∙f_t_^−1^. f_t_* can be expressed as *f_t_ = 0.447 + 1.4034exp(−T/26.815)* (adapted from [15] using corrected formula from [14]).

## 3 Design files summary

The hardware files needed to reproduce the sample holder and software files used to carry out measurements are listed in Table 1.

**Table 1:** List of design files.

Design file name	File type	Open source license	Location of the file (DOI)
electrode.dxf	DXF file	CC BY 4.0	https://doi.org/10.17605/OSF.IO/9AYVT
top.step	STEP file	CC BY 4.0	https://doi.org/10.17605/OSF.IO/9AYVT
top_cap.step	STEP file	CC BY 4.0	https://doi.org/10.17605/OSF.IO/9AYVT
bottom.step	STEP file	CC BY 4.0	https://doi.org/10.17605/OSF.IO/9AYVT
bottom_cap.step	STEP file	CC BY 4.0	https://doi.org/10.17605/OSF.IO/9AYVT
sgf_8slot_spread.xml	XML file	CC BY 4.0	https://doi.org/10.17605/OSF.IO/9AYVT
sgf_8slot_protocol.xml	XML file	CC BY 4.0	https://doi.org/10.17605/OSF.IO/9AYVT
sgf_settings.xml	XML file	CC BY 4.0	https://doi.org/10.17605/OSF.IO/9AYVT
Temperature logger configuration	Directory with temperature logger scripts	CC BY 4.0	https://doi.org/10.17605/OSF.IO/9AYVT
Data processing	Directory with data and data processing scripts	CC BY 4.0	https://doi.org/10.17605/OSF.IO/9AYVT

The design files are divided into four categories:

### 3.1 Hardware design

• *electrode.dxf*: 2D schematic for electrodes, for laser cutting from a 5 mm stainless steel plate.
• *top.step & bottom.step*: 3D schematics, for 3D printing of main lid bodies.
• *top_cap.step & bottom_cap.step*: 3D schematics, for 3D printing of caps that seal the lid’s inner compartment.

### 3.2 Terrameter LS2 configuration (editable with text editor)

• *sgf_8slot_spread.xml: description of relative electrode layout.*
• *sgf_8slot_protocol.xml: query sequence defining a list of the electrode quadrupoles used during measurement.*
• *sgf_settings.xml: measurement settings.*

### 3.3 Temperature logger configuration

• Directory containing scripts for controlling Comet W0741 temperature logger. Refer to the readme.md for instructions on how to set up and run temperature measurements.

### 3.4 Data processing

• Directory containing R scripts for data processing and example data. Refer to the file readme.md for instructions on how to set up and run the data processing scripts. The scripts require version 4.2.1 (“Funny-Looking Kid”) of R and are intended for use with the software RStudio (https://posit.co/downloads/).

## 4 Bill of materials summary

The components used to assemble the sample holder are listed in Table 2. The total cost for a system of 8 sample holders with an ABEM Terrameter LS2 resistivity meter was 57900$. Excluding the resistivity meter the cost was 7900$. An ABEM Terrameter LS2 is very costly in relation to the other components, many times more than the rest of the components together, and as it is primarily designed for time- and cost-efficient field scale measurements under challenging conditions it is in several ways overqualified for this application. Although the high-quality input section of that instrument is valuable to ascertain good quality data, it should be possible to use other resistivity meters instead. One alternative may be the open-source and open-hardware OhmPi instrument [16] which costs approximately 5000€, although the spread and protocol files as well as the measurement scripts would need to be adapted for it. Furthermore, the measurement accuracy is likely to be affected, due to lower resolution ADC (analogue to digital converter) among other differences in measurement hardware and software design, and the data quality would need to be assessed to verify that it meets the requirements of the intended application.

**Table 2:** Bill of materials.

Designator	Component	Qty	Unit cost	Total cost	Source of materials	Material type
Top lid body	SLS-prototype/3D print	8	$28	$224	GT Prototyper AB/gtp.se	Polymer
Bottom lid body	SLS-prototype/3D print	8	$28	$224	GT Prototyper AB/gtp.se	Polymer
Top lid cap	SLS-prototype/3D print	8	$28	$224	GT Prototyper AB/gtp.se	Polymer
Bottom lid cap	SLS-prototype/3D print	8	$28	$224	GT Prototyper AB/gtp.se	Polymer
Impregnation agent	Dichtol coating for SLS-prototype/3D print	1	$60	$60	GT Prototyper AB/gtp.se	Polymer
Electrodes	Stainless steel electrodes	64	$4	$256	KPMV/https://www.kpmv.se	Metal
Ring terminal	Terminals RING 18–14 8 Cut Strip	64	$0	$13	TE Connectivity/mouser.com	Metal
Ring seal	O-RING. M SEALS 55.0X3.0 NBR 7 0	16	$1	$19	Ahlsell/ahlsell.se	Polymer
Vent hole screw	Hex screw with cylindrical head 10 mm	16	$1	$8	Ahlsell/ahlsell.se	Metal
Temperature sensor	PT 1000 NB-PTCO-157	16	$3	$48	TE Connectivity/rs-online.com	Composite
Electrode cable 1	SAC-4P-M12MS/ 1,5–800	16	$19	$304	*Phoenix* Contact/phoenixcontact.com	Composite
Electrode cable 2	SAC-4P- 1,5-PUR/M12FS	16	$19	$304	*Phoenix* Contact/phoenixcontact.com	Composite
Connector dust cap	PROT-M12 FS-PA-CHAIN	16	$3	$48	*Phoenix* Contact/phoenixcontact.com	Composite
Temperature sensor cable	Unitronic PUR 4x 0.34 mm^2^ sensor cable	16	$3	$48	LAPP/elfa.se	Composite
Temperature cable connector	Cable Mount RCA Plug, gold plated	16	$2	$32	RS Pro/rs-online.com	Composite
Cable gland	AG 12LSR GY4-5	32	$2	$64	RS Pro/rs-online.com	Polymer
Potting agent	Black PUR Potting Compound 8810-375ML	2	$39	$78	MG Chemical/rs-online.com	Polymer
Glue	Ethyl cyanoacrylate	2	$9	$18	loctiteproducts.com	Polymer
32-pin cable connector	Amphenol Socapex 45106A1832PPG50	2	$111	$222	Amphenol/rs-online.com	Composite
Wi-Fi router	RT-AX58U	1	$102	$102	Asus.com	Other
UPS battery	fit-Uptime	1	$47	$47	fit-iot.com	Other
Mini-PC	Fitlet2	1	$600	$600	fit-iot.com	Other
Temperature logger	Comet W0741	8	$590	$4 720	Comet System/cometsystem.se	Other
Resistivity meter	ABEM Terrameter LS2	1	$50 000	$50 000	Guideline Geo AB/	Other

## 5 Build instructions

### 5.1 Overview

The full set-up consists of several different parts (listed in Table 2), with some being off the shelf and others being proprietary. All the different components are shown on the image in Fig. 2. The eight sample holder lids and the cable assemblies are the components which need to be built. Each sample holder (shown in Fig. 1) consists of a top and bottom lid while each cable assembly is a gathering of several 4 conductor cables into a 32-pin connector of the *Resistivity meter*.

Before assembly it is necessary to prepare the following components: *Top lid, Bottom lid, Top lid cap, Bottom lid cap* and the *Electrodes*. The lids and lid caps need to be SLS 3D printed from the respective STEP-files (Table 1) and treated with the impregnation agent for water resistance. The *Electrodes* should be laser cut from 5 mm stainless steel, using *electrode.dxf*, and each have a *Ring terminal* spot welded to the centre of the main surface. The *Ring terminals* could be exchanged to any other ring terminal of a similar size which can withstand the spot welding.

### 5.2 32-pin cable connector

For each of the two cable assemblies used in connecting to the Resistivity meter the following components are needed:

• 1 *32-pin cable connector*
• 8 *Electrode cable 2*.

The following steps describe the production of the *32-pin cable connector*:

1. Thread the 8 *Electrode sensor cable 2* assemblies through the outer housing of the *32-pin cable connector*.
2. Remove the outer 4 cm of insulation from each *Electrode cable 2* assembly and feed the individual conductors through the insulator (included with the *32-pin cable connector*). The wiring of the *Electrode cable 2* is done in ascending order to the alphabetical order on the *32-pin cable connector* (see Appendix A).
3. Remove the first 0.5 cm of insulation from the conductors of the *Electrode cable 2* and solder them to the pins of *32-pin cable connector*. The order of soldering varies depending on preference but pay attention to using the correct pin connections. For simplicity, the *Electrode cable 2* can, at this point, be individually labelled with 1–8. This labelling is done according to the order they are soldered to the *32-pin cable connector*. Labelling at this point can avoid extra work for the same process later.
4. Close up the *32-pin cable connector* and repeat for the second set of materials.

### 5.3 Top lids

The assembly of each top lid requires the following:

• *Top lid body*
• *Top lid cap*
• 4 *Electrodes* (with *Ring terminals*)
• 1 *Ring seal*
• 1 *Vent hole screw*
• 1 *Temperature sensor*
• 1 *Electrode sensor cable assembly*
• 1 *Connector dust cap*
• 1 *Temperature sensor cable*
• 1 *Temperature cable connector*
• 2 *Cable glands*
• *Potting agent*
• *Glue*

The following steps should be followed to create the top lid:

1. Mount a *Cable gland* in each of the two holes of the *Top lid cap*, with the gland on the flat side, and thread the *Electrode cable 1* through one of them from the flat side of the cap.
2. Remove the outer insulation from the first 5 cm of the *Electrode cable 1*. Feed the *Electrode cable 1* through one of the internal holes of the *Top lid body* and feed each individual conductor through its respective hole for the electrodes. Make sure that the conductors go through the proper holes according to the wiring schematics found in Appendix B.
3. Remove the insulation from 1 cm of each conductor and crimp an electrode on each one. Depending on the chosen Ring terminal it can be advantageous to add some solder to the connection for strength and better electrical connection.
4. Add *Glue* to the edge of the *Top lid body* where the electrodes will rest and glue the electrodes in place. Make sure to remove any excess glue which gets on the electrodes outside surface to avoid contact problems in application. The *Glue* will later serve as a seal for the potting agent so make sure the *Glue* is added along the full perimeter of the electrode.
5. Solder the *Temperature sensor* to the *Temperature sensor cable* with each leg connected to 2 conductors making sure to keep track of which conductors are paired up,
6. Feed the *Temperature sensor* and the cable through the free internal hole in the *Top lid body* and *glue* the sensor down in the indentation in the centre between the electrode holes.
7. Feed the other end of the *Temperature sensor cable* back through the *Top lid cap* using the free *Cable gland* from the nut side. Solder the *Temperature cable connector* to the end of the cable with two conductors on each pin. Make sure that the same conductors are paired up on the pins of the connector as well as on the *Temperature sensor* in step 5.
8. The top lid is now ready for filling with *Potting agent*. This filling process is better done in a big batch, so prepare all Top lids and Bottom lids up to this step before mixing the *Potting agent*.
9. Mix the *Potting agent* and fill the *Top lid body* to around the internal holes and let it cure.
10. After the *Potting mix* has cured the Top lid can be closed with the *Top lid cap*, making sure to tighten the *Cable gland* around the cables.
11. Add the *Vent hole screw* to the small vent hole on the side of the top lid, add the *Ring seal* to the groove on the electrode side of the top lid and add the *Connector dust cap* to the *Electrode cable 1*.

### 5.4 Bottom lids

The assembly of each bottom lid requires the following:

• *Bottom lid body*
• *Bottom lid cap*
• 4 *Electrodes* (With *Ring terminals*)
• 1 *Ring seal*
• 1 *Vent hole screw*
• 1 *Temperature sensor*
• 1 *Electrode cable 2*
• 1 *Connector dust cap*
• 1 *Temperature sensor cable*
• 1 *Temperature cable connector*
• 2 *Cable gland*
• *Potting agent*
• *Glue*

The following steps should be followed to create the bottom:

1. Mount *Cable gland* in the two holes of the *Bottom lid body* and thread the *Electrode cable 1* through one of them from the outside side.
2. Remove the outer insulation from the first 5 cm of the *Electrode cable 1*, feed each of the individual conductors through the respective holes for the electrodes. Make sure that the conductors go through the proper holes according to the wiring schematics found in Appendix B.
3. Remove the insulation from 1 cm of each conductor and crimp an *Electrode* on each one. Depending on the chosen *Ring terminal* it can be advantageous to add some solder to the connection for strength and better electrical connection.
4. Add *Glue* to the edge of the *Bottom lid body* where the *Electrodes* will rest and glue the electrodes in place. Make sure to remove any excess glue which gets on the electrodes outside surface to avoid contact problems in application. The glue will later serve as a seal for the potting agent so make sure it is added along the full perimeter of the electrode.
5. Solder the *Temperature sensor* to the *Temperature sensor cable* with each leg connected to 2 conductors making sure to keep track of which conductors are paired up,
6. *Glue* the *Temperature sensor* down in the indentation in the centre between the electrodes.

Feed the other end of the *Temperature sensor cable* back out the *Bottom lid body* through the free *Cable gland*.

7. Solder the *Temperature cable connector* to the free end of the *Temperature sensor cable*. Make sure that the same conductors are paired up on the pins of the connector as were paired on the *Temperature sensor* in step 5.
8. Tighten *Cable gland* for strain relief and moisture protection.
9. The bottom lid is now ready for filling with *Potting agent*. This process is better done in a big batch, so prepare all top lids and bottom lids up to this step before mixing the *Potting agent*.
10. Mix the *Potting agent* and fill the *Bottom lid body* to well above the *Ring terminals* and let it cure.
11. After the *Potting mix* has cured, glue the *Bottom lid cap* in place in the matching recess in the *Bottom lid body*.
12. Add the *Vent hole screw* to the small vent hole on the side of the *Bottom lid*, add the *Ring seal* to the groove on the electrode side of the bottom lid and add the *Connector dust cap* to the Electrode sensor cable assembly.

**Fig. 4:**
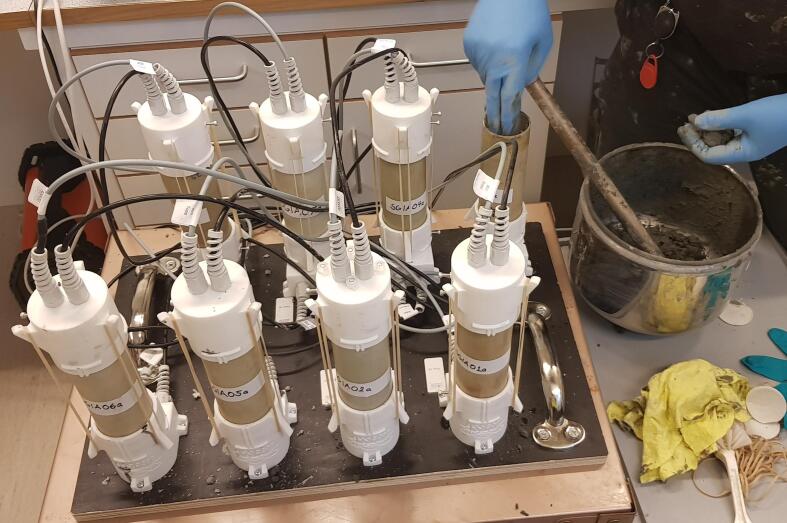
A set of eight samples with attached sensor lids. The upper right sample is being prepared by packing lime-cement mixture into a sample cylinder. The bottom lids of the samples are mounted onto a wooden board for stability.

The bottom lid design includes 3 holes for screws that can be used to mount the lids onto e.g. a wooden board (see Fig. 4).

### 5.5 Safety precautions

In the assembly process safety precautions need to be taken in the cases of hot work and fumes. For hot work involving laser cutting of the *Electrodes*, spot welding of the *Ring terminals* and soldering of electronics, it is important to use appropriate safety measures. A well-ventilated area and suitable respiratory protection wear should be used for work with soldering and the solvents used in the Impregnation agent and Potting agent.

## 6 Operation instructions

### 6.1 Overview

The system consists of the components shown in Fig. 2. The lids’ electrode cables connect, through the 32-pin cable connector adapter, to the resistivity meter. The lids’ temperature cables connect to the temperature loggers. Both the resistivity meter and the temperature loggers are connected to the Mini-PC via the router. This chapter describes the set-up of the components and how to measure data. It also describes the sample preparation methodology used for the data presented in Chapter 7.

### 6.2 Resistivity meter, temperature logger preparation

Always make sure that the resistivity meter’s red emergency stop button is pressed before handling hardware. Prepare the resistivity meter for measurement using the spread, protocol and settings files as described in the Terrameter LS2 manual [17].

Prepare the temperature logger(s) for measurement as described in the Comet Temperature Sensor manual [18]. Fig. 2 shows the system setup.

The router is used to connect the resistivity meter (via cable) and temperature loggers (wirelessly) to the Mini-PC in a local network. The Mini PC is used to control the instruments, and can be set up to automate measurements if desired (refer to [19], paper 4, for Terrameter LS2 automation code example).

### 6.3 Sample preparation

For other types of sample material practical details may vary. For the measurement data presented in the Chapter 7 test case, lime-cement samples were used (Fig. 4). The samples were prepared by lab technicians in the Swedish Geotechnical Institute lab in accordance with standard methodology [20], [21]. The lime-cement mixture consisted of clay mixed with a lime-cement binder. A marine deposited clay obtained from a field site in Gothenburg was used. Before mixing, the clay had a water content of 62.9%. The binder consisted of lime and cement at a 3:7 mass ratio. A mass of binder was added to the clay equivalent to 60 kg binder per 1 m3 of clay.

The mixture was mixed mechanically into a homogeneous mass and then filled by hand into sample cylinders as seen in Fig. 4. A rod was used to tamp the mixture and remove air bubbles. Since this methodology produces a material of high homogeneity, sample heterogeneity was not expected to affect measured resistivities to a significant degree (discussed further in Chapter 7).

### 6.4 Lid attachment

Attach both lids to each sample cylinder, making sure lids are inserted fully. If necessary, apply lubricant to the outer surface of the sample cylinder.

The valve screws on the lids can be opened as needed to release air and liquid between sample material and electrodes.

Lime-cement samples dry and harden over time during curing. This may cause poor contact between the electrode and sample material. To improve the electrode contact resistance, wetted cloth was placed between the electrodes and sample material. Additionally, long elastic bands (as seen in Fig. 4) can be added to the three notches on each side of the lids and bottoms to ensure electrode contact. Align the bottom and top lids’ T-shaped notches and valve screws relative to each other (as in Fig. 4), by rotating the top lid.

Connect the lids’ cables to the resistivity meter and the temperature logger. It is important to note which input connection on the instruments that a sample is attached to. It is recommended to enumerate samples in the same way as the instrument sockets (e.g. plug “Sample1” into “Socket1”). The lids’ exteriors have T-shaped notches that correspond to the sample’s extent. Measure sample length by measuring the distance between the horizontal notch on the top and bottom lid using callipers (sample length is needed for correct calculation of resistivity). Enter sample names and measured sample lengths into the excel file template.

### 6.5 Starting measurements

Make sure the instrument clocks are correct and synchronized, and to measure temperature data overlapping the resistivity measurement period for temperature compensation.

After connecting the instrument setup and preparing the samples, release the stop button on the resistivity meter. Start temperature measurements by running the python script as described in the *readme* file and then start the resistivity measurements.

### 6.6 Data processing

The processing scripts are prepared to recreate the results in Chapter 7, using with the sample data provided. Refer to the *readme* file for instructions on how to set up and run the scripts.

### 6.7 Calibration

Calibration of the sensor lids was done using liquid samples with a known resistivity of ρ_25°C_ = 7.08 Ωm. The resulting geometric factor, normalized with regards to sample length, was *k*⋅*L_sample_ =* 2.54⋅10^−3^ m. For the measurements shown in Chapter 7, *k* values for calculation of resistivities were derived by dividing *k*⋅*L* with the respective sample lengths.

## 7 Validation and characterization

As a test case for the sample holder system, the resistivity of 8 laboratory-prepared lime-cement samples was measured during curing. The list below describes the test characteristics:

• Sample material: Lime-cement mixture (described in Chapter 6)
• Sample dimensions: 25.0 mm radius, 153.1–165.9 mm (154 mm average) length
• DC current: 1 mA
• Received potential range: 1⋅10^−2^ to 1⋅10^−1^ V
• Temperature range: 18.7 to 22.3°C
• Average coefficient of variation: 0,17%±0.0033 (95% confidence interval)
• Electrode contact resistance range: 10^2^ to 10^3^ Ω
• Test duration: 93 days
• # measurements: 39,200 data points

### 7.1 Data description

**Fig. 5:**
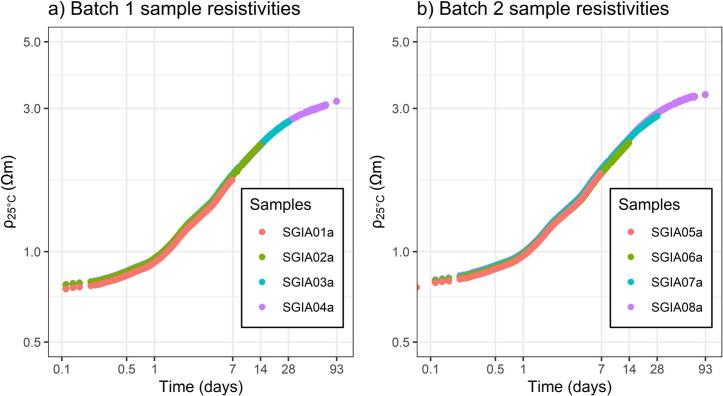
The graphs show temperature-compensated resistivity (ρ_25°C_) over time of eight lime cement samples. Batch 1 (a) and 2 (b) used the same lime cement recipe but were mixed separately, producing four samples from each batch. All samples were measured from time 0 days, overlapping shorter measurement series are shown on top of the longer ones.

Fig. 5 shows the resistivity change during hydration of lime-cement samples that were prepared in two separate batches. The resistivity measurements were automated to collect data repeatedly during a period of 93 days. Samples were removed and opened at the intervals marked on the time axis (e.g. SGIA01a and SGIA05a were measured until time 6.9, when they were removed). A malfunction of the resistivity meter caused the gaps that can be seen in the later measurements (samples SGIA04a and SGIA08a). Resistivities increased over time as the samples hardened. Small resistivity differences between samples (e.g. SGIA07a and SGIA08a resistivities at 27.9 days) may be due to the packing of individual samples, material inhomogeneities or error in sample length measurements. For length errors that are small in relation to total sample length, the resulting resistivity error is proportional to the length error. E.g. for a sample length of 155 mm, a measurement error of ± 5 mm (i.e. ± 3.2%) results in a resistivity error of ± 3.2%.

**Fig. 6:**
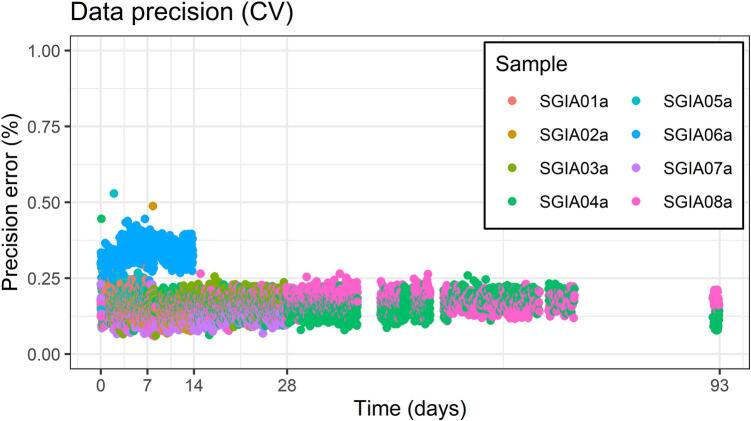
Measurement precision (CV − coefficient of variation in %) over time for each sample and measurement occasion.

Fig. 6 shows precision error over time, which stayed consistently low throughout the test period. The small spike around day 7 coincides with when sample SGIA01a and SGIA05a were removed and may be due to accidentally disturbing the other samples, affecting the electrode contact. Sample SGIA06a (blue circles) shows a marginally higher error which may be due to the sample’s somewhat higher contact resistances.

Precision error was estimated by analysing the variation between data from the same measurement occasion, resulting in an average coefficient of variation $\overset{¯}{\mathrm{CV}}$ of 0,17%±0.001. For each sample and measurement occasion, several rotations of the same geometric factor were measured by varying the electrodes used. In theory, an arbitrary sample, homogeneous or not, should give the same measurement value for the reciprocal pairs. For homogeneous samples all rotations should give the same result. By evaluating the variability between these rotations, the effect of sample heterogeneity is incorporated into the error. The coefficient of variation (CV) of each measurement group was calculated as $\mathrm{CV}=100∙\sigma /\overset{¯}{x}$. To obtain the standard deviation σ used to calculate CV, the combined standard deviation for a group of rotations was calculated from stacked measurements using Eq. (1):

(1) $$\sigma =\sqrt{\frac{\sum {(n}_{i}-1)∙\sigma_{i}^{2}+\sum n_{i}{\left({\overset{¯}{x}}_{i}-\overset{¯}{x}\right)}^{2}}{\left(\sum n_{i}\right)-1}}$$

Formula for combined standard deviation σ of all rotated measurements in a group (i.e. belonging to the same occasion and sample), where n_i_ is measurement stack size, σ_i_ is standard deviation of a measurement stack, $\overset{¯}{x_{i}}$ is stack mean value, and $\overset{¯}{x}$ is the group combined mean.

Since sample resistivities changed between measurement occasions it was not meaningful to calculate a combined CV for the overall 93-day period. To give an indication of the overall precision error, an average $\overset{¯}{\mathrm{CV}}$ was calculated from the CV’s of all measurement groups. The margin of error (ME) with a 95% confidence interval was calculated using the formula $\mathrm{ME}=\pm t∙\left(\sigma_{\mathrm{CV}}/\sqrt{n}\right)$, where n was 9840 (the number of measurement groups), σ_CV_ was the standard deviation between measurement groups, and a t value of 1.96 was used.

To compare the repeatability between our sample holder design and a conventional soil box, a 7.08 Ωm (at 25 °C) sample solution was measured over a period of 3 days. The measurements were made under the same temperature conditions and using the same Terrameter LS2 resistivity meter and sample solution. Lime-cement samples were not used for this comparison due to the impracticalities of using a conventional soil box with lime-cement material and there were concerns of damaging the soil-box during sample removal.

For these measurements the CV was 0.92% for the soil box and 0.57% for our sample holder design. These values are in the same range as those obtained from the measurements of lime-cement (shown in Fig. 6). Using a geometric factor (*k)* calibrated for each individual sample holder, the average resistivities are very close to the nominal 7.08 Ωm of the reference solution; 7.08 ± 0.04 Ωm for our sample holder design and 7.08 ± 0.10 Ωm for the soil box. To avoid individual calibration for the *k* of each sample holder (which is impractical for a full set-up with 8 sample holders), a general *k* can be used. Normalizing for sample length (as described in Chapter 2) also accounts for variations between individual sample lengths. A *k*⋅*L* value of 2.54⋅10^−3^ m (obtained from measurement values from multiple sample holder copies) results in an average resistivity of 7.08 ± 0.4 Ωm. This range can be taken as an estimate of the design’s accuracy (reflecting tolerances of the parts and the described assembly process).

**Fig. 7:**
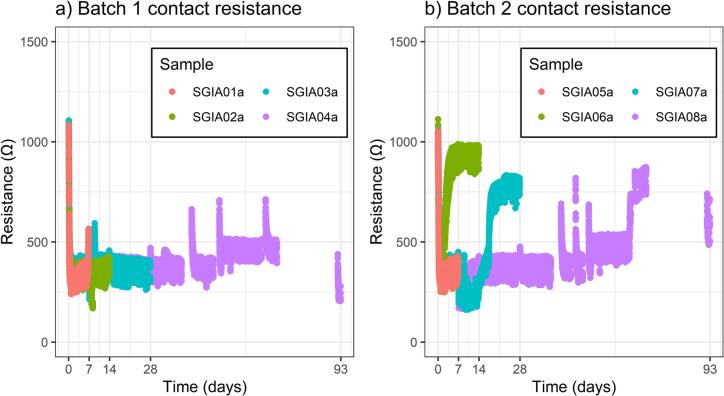
Electrode contact resistance values over time for the samples in Batch 1 (a) and Batch 2 (b).

Electrode contact resistance was estimated by using one electrode as current sink and the remaining seven electrodes as current source, i.e. the focus-one electrode test method [22]. It was assumed that the contact resistance contributed from the source electrodes would be small (due to their larger combined contact area) in relation to that of the sink electrode, and the value would thus give an approximation of the current sink electrode’s contact resistance. By varying electrode source and sink combinations, a contact resistance value for each electrode was measured in this way. This procedure was done repeatedly during the 93-day measurement period for all samples. Fig. 7 shows the measured electrode contact resistances. The values vary in the range of 10^2^ to 10^3^ Ω. All samples show a fast initial decrease in resistances which may be related to changes in the pore fluid chemistry during early hydration of the lime-cement. Despite the drying of the lime-cement that occurs over time, only samples SGIA06a and SGIA07a show a gradual increase in contact resistance. Most samples show temporary spikes in contact resistance (e.g. sample SGIA01a at 7 days). These spikes occur at times when manual work with the samples took place (e.g. removal of samples and sample length measurements) and are likely a result of accidental disturbances to the sample holders. The variations in contact resistances between samples do not meaningfully affect the measured resistivities which are very similar to each other as seen in Fig. 5, and measurement error is consistently low (Fig. 6).

**Fig. 8:**
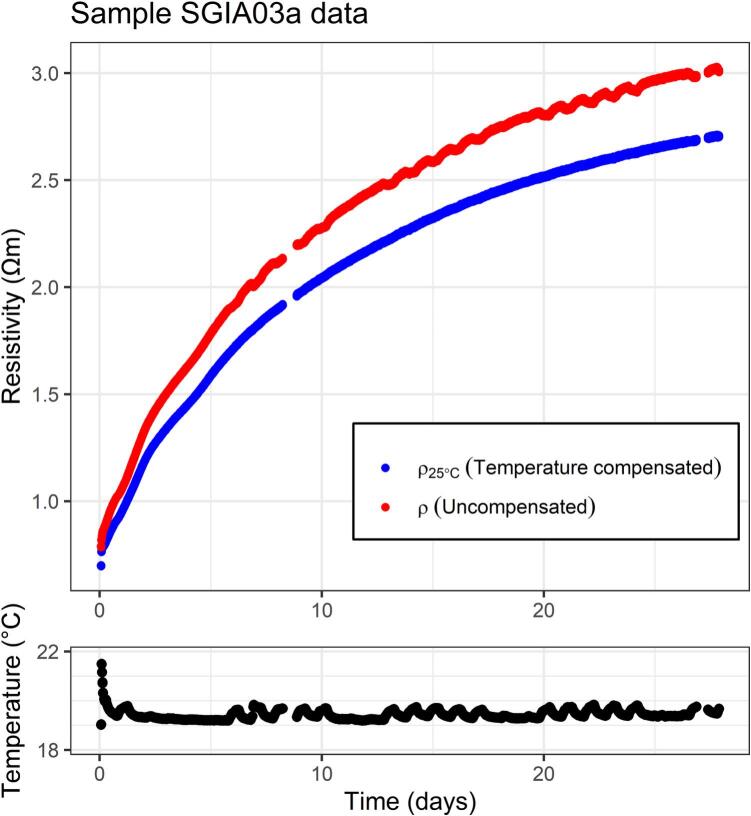
The top graph shows temperature compensated (ρ_25°C_) and uncompensated (ρ) resistivity over time, the bottom graph shows the corresponding temperature.

Fig. 8 shows the effect of temperature variations on resistivity for sample SGIA03a. The heat generated by the setting lime-cement samples causes an initial temperature spike at the start of the measurements, when the hydration reaction rate is highest. As a result of this the uncompensated resistivity (ρ) shows a slightly different trend compared to the temperature compensated resistivity (ρ_25°C_). Due to the small mass of the sample, sample temperature quickly aligns with the environment. The laboratory's ventilation cycle during weekdays can be seen as a recurring pattern in the uncompensated resistivity curve.

In conclusion our sample holder design enables temperature compensated four-electrode resistivity measurements with non-intrusive sensors. It was tested for the use case of laboratory-prepared lime-cement samples and the sample holders were shown to provide large amounts of high-precision resistivity data ($\overset{¯}{\mathrm{CV}}$ = 0.17%) over an extended measurement period (93 days). Environmental temperature effects on sample resistivity were compensated for using data from the integrated temperature sensors. The effect of sample heterogeneities was not separated from other error sources in this study. However, a comparison of the small differences in measured resistivities between samples (together with an evaluation of measurement precision) indicates that the compound effect of sample heterogeneity and other error sources was small. Lime-cement was the tested use case for our sample holder design, but it should perform similarly with other stabilized soft soil mixtures. A practical application is shown by [12], where our sample holder design was used to compare the resistivity and unconfined compressive strength of dredge mass stabilization samples.

The dimensions of the sample holder also enable the capability to measure field samples acquired with a geotechnical rig, though this capability has not been verified in this study and needs to be tested in future work. The performance in coarser soils and in unsaturated conditions has also not been tested, and a sensitivity study of error sources should be made for use cases where e.g. significant heterogeneity or length variation is expected. By varying the electrode configuration, other arrays than the one used here are possible. This could be utilized to e.g. provide information about sample heterogeneity.

## CRediT authorship contribution statement

**Mikael Lumetzberger:** Writing – original draft, Visualization, Software, Methodology, Investigation, Conceptualization. **Torleif Dahlin:** Writing – review & editing, Supervision, Project administration, Methodology, Conceptualization. **Tina Martin:** Writing – review & editing, Supervision. **Per Hedblom:** Software, Methodology, Investigation, Conceptualization. **Simon Rejkjær:** Writing – original draft, Methodology, Investigation. **Per-Ivar Olsson:** Supervision, Methodology, Conceptualization.

## Declaration of competing interest

The authors declare that they have no known competing financial interests or personal relationships that could have appeared to influence the work reported in this paper.

## Figures and Tables

**Table:** 

Index	32 pin connector pin	Electrode cable 2 pin	4-pin cable naming
1	A	1	1
2	B	2	
3	C	3	
4	D	4	
5	E	1	2
6	F	2	
7	G	3	
8	H	4	
9	J	1	3
10	K	2	
11	L	3	
12	M	4	
13	N	1	4
14	P	2	
15	R	3	
16	S	4	
17	T	1	5
18	U	2	
19	V	3	
20	W	4	
21	X	1	6
22	Y	2	
23	Z	3	
24	a	4	
25	b	1	7
26	c	2	
27	d	3	
28	e	4	
29	f	1	8
30	g	2	
31	h	3	
32	i	4	

**Figure:**
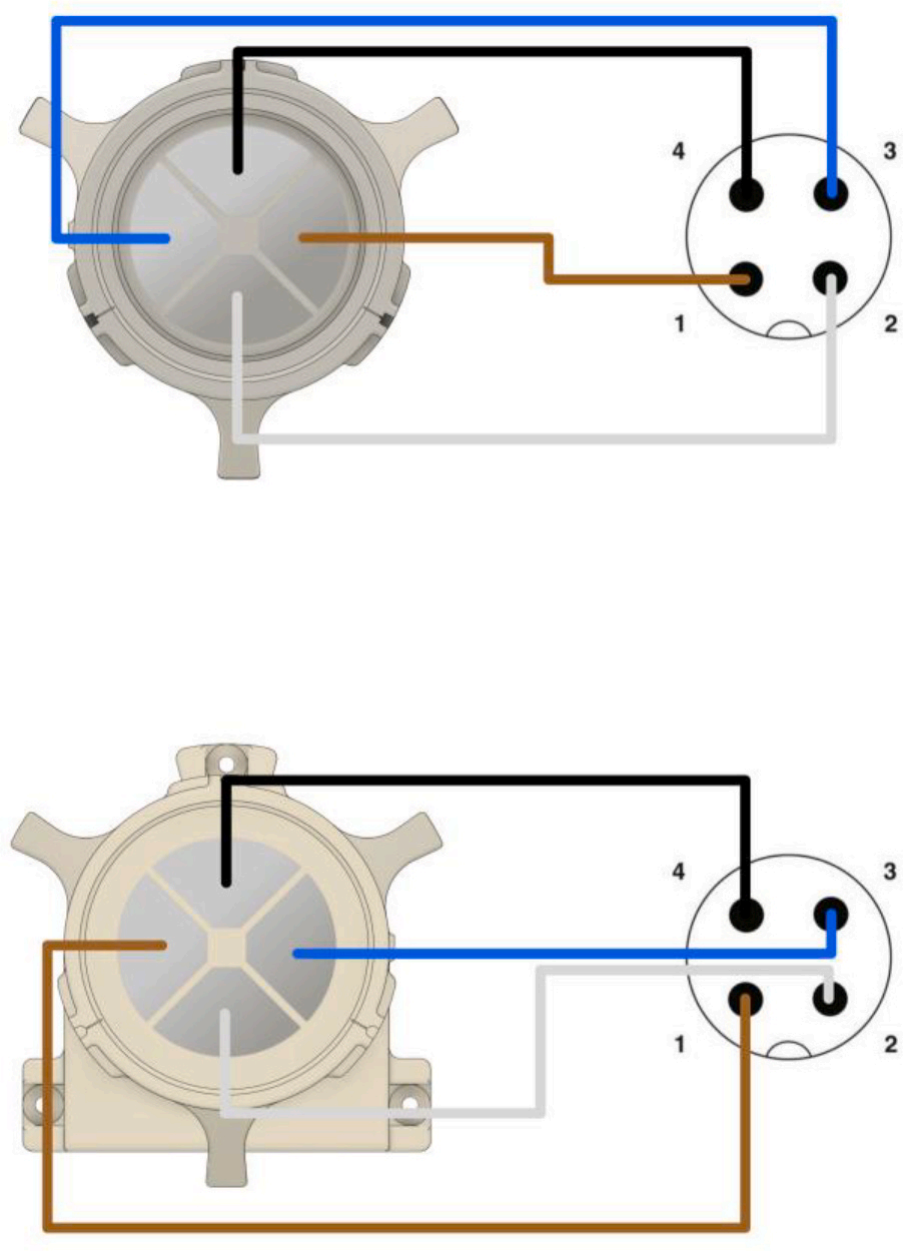


## Appendix A 32-pin connector wiring

**Table A1.** 32-pin connector wiring.
